# Biomechanical Modeling, Muscle Synergy-Based Rehabilitation Assessment, and Real-Time Fatigue Monitoring for Piano-Integrated Upper Limb Therapy

**DOI:** 10.3390/biomimetics10070419

**Published:** 2025-06-29

**Authors:** Xin Zhao, Ying Zhang, Yi Zhang, Shuo Jiang, Peng Zhang, Jinxu Yu, Shuai Yuan

**Affiliations:** 1 School of Arts and Design, Yanshan University, Haigang District, Qinhuangdao 066000, China; cindy@ysu.edu.cn; 2 Arts Department of Qinhuangdao Vocational and Technical College, Beidaihe District, Qinhuangdao 066100, China; zhangying@qvc.edu.cn; 3 Organization and Publicity Office of the CPC Qinhuangdao Vocational and Technical College Committee, Beidaihe District, Qinhuangdao 066100, China; zhangyi@qvc.edu.cn; 4 Department of Design, Kyungpook National University, Daegu 41566, Republic of Korea; jshuo1024@knu.ac.kr (S.J.); 2021327350@knu.ac.kr (P.Z.); 5 College of Mechanical and Automotive Engineering, Ningbo University of Technology, Ningbo 315211, China; jinxuyu@nbut.edu.cn

**Keywords:** piano-based rehabilitation, biomechanical modeling, muscle synergy analysis, non-negative tensor factorization (NTF)

## Abstract

Piano-based occupational therapy has emerged as an engaging and effective rehabilitation strategy for improving upper limb motor functions. However, a lack of comprehensive biomechanical modeling, objective rehabilitation assessment, and real-time fatigue monitoring has limited its clinical optimization. This study developed a comprehensive “key–finger–exoskeleton” biomechanical model based on Hill-type muscle dynamics and rigid-body kinematics. A three-dimensional muscle synergy analysis method using non-negative tensor factorization (NTF) was proposed to quantitatively assess rehabilitation effectiveness. Furthermore, a real-time Comprehensive Muscle Fatigue Index (CMFI) based on multi-muscle coordination was designed for fatigue monitoring during therapy. Experimental validations demonstrated that the biomechanical model accurately predicted interaction forces during piano-playing tasks. After three weeks of therapy, patients exhibited increased synergy modes and significantly improved similarities with healthy subjects across spatial, temporal, and frequency domains, particularly in the temporal domain. The CMFI showed strong correlation (r > 0.83, *p* < 0.001) with subjective fatigue ratings, confirming its effectiveness in real-time fatigue assessment and training adjustment. The integration of biomechanical modeling, synergy-based rehabilitation evaluation, and real-time fatigue monitoring offers an objective, quantitative framework for optimizing piano-based rehabilitation. These findings provide important foundations for developing intelligent, adaptive rehabilitation systems.

## 1. Introduction

Upper limb impairments due to stroke or neurological injuries significantly hinder patients’ ability to perform daily activities, underscoring the necessity for effective rehabilitation strategies [1,2]. In recent years, task-oriented rehabilitation devices integrating functional activities, such as piano-playing tasks, have emerged as promising therapeutic approaches, leveraging patient engagement and promoting neuroplasticity [3,4]. Previously, we developed a novel upper limb rehabilitation device combining piano playing with occupational therapy to enhance motor recovery in patients with hand dysfunctions [5]. Preliminary evaluations demonstrated positive outcomes regarding patient engagement and functional recovery. Nevertheless, critical gaps remain, specifically regarding biomechanical understanding, quantitative assessment methods, and fatigue monitoring during therapy, warranting further comprehensive investigation.

Accurate biomechanical modeling is crucial for enhancing rehabilitation device effectiveness, ensuring physiological alignment with human hand anatomy and kinematics [6]. Traditional hand rehabilitation devices often neglect detailed biomechanical interactions among the hand, assistive device, and therapeutic tasks, potentially leading to inefficient rehabilitation or unintended strain on patients’ joints and muscles [7,8]. Previous research underscores the importance of employing comprehensive biomechanical models, including musculoskeletal dynamics and joint force interactions, to optimize therapeutic interventions [9,10]. Specifically, Hill-type muscle models have been recognized for their effectiveness in accurately simulating human muscle behavior during dynamic tasks, thus providing valuable insights for rehabilitation device design and evaluation [11,12]. However, detailed biomechanical analyses of hand interactions with piano-based rehabilitation devices remain scarce, limiting our understanding of therapeutic mechanisms and optimization opportunities.

In addition to biomechanical considerations, precise evaluation of rehabilitation outcomes presents significant challenges, particularly when assessing complex motor tasks like piano playing. Conventional clinical assessments (e.g., Fugl–Meyer Assessment or the Box-and-Block Test) often lack sensitivity in capturing subtle improvements specific to task-oriented therapy [13,14]. Recent advancements in electromyography (EMG)-based muscle synergy analyses, particularly using non-negative matrix factorization (NMF), offer sophisticated methodologies to quantify muscle coordination patterns during rehabilitation tasks [15,16]. Muscle synergy analysis can objectively capture changes in neuromuscular control strategies, providing detailed metrics to evaluate rehabilitation efficacy more sensitively than traditional assessments [17]. Despite these advancements, applications of muscle synergy-based evaluations to piano-integrated rehabilitation remain underexplored. Traditional muscle synergy analysis methods typically operate in two dimensions, focusing on time and space (time × space) [15]. While this approach provides valuable insights into muscle coordination patterns, it does not fully capture the frequency domain characteristics of the EMG signals, which are important for tasks involving rapid, precise muscle activation, such as piano playing. The absence of frequency domain analysis limits the ability to assess muscle fatigue and fine motor control, both of which are critical in rehabilitation tasks that require high levels of precision.

Moreover, fatigue is a critical yet frequently overlooked aspect during repetitive rehabilitation exercises. Excessive fatigue negatively impacts motor performance, learning efficiency, and patient safety [18,19]. Traditional subjective measures like perceived exertion scales lack reliability, especially in neurologically impaired populations [20,21]. Recent studies demonstrate that EMG-based fatigue indicators—such as frequency spectrum shifts—can provide objective, real-time fatigue assessments, enabling dynamic adaptation of rehabilitation intensity [22,23]. However, integration of EMG-based fatigue monitoring into upper limb rehabilitation devices, especially those involving task-oriented activities like piano playing, remains limited.

While existing biomechanical models, such as the Hill-type muscle model, have been widely used for muscle dynamics simulations, they are often designed for isolated muscles or joints and do not account for the complex multi-joint interactions required in task-oriented rehabilitation activities [11]. Traditional biomechanical models may not accurately capture the dynamic interactions between the multiple finger joints and the piano keys, which are critical for piano-based rehabilitation. Our proposed biomechanical model integrates these interactions, providing a more comprehensive and accurate representation of the rehabilitation task.

Similarly, traditional fatigue-monitoring methods, such as EMG spectral analysis, have limitations in their ability to capture the real-time dynamics of muscle fatigue during complex tasks like piano playing. EMG spectral analysis typically focuses on single-muscle fatigue, but rehabilitation tasks like piano playing require multi-muscle coordination that is not fully captured by these methods [15,20]. In contrast, our Comprehensive Muscle Fatigue Index (CMFI) takes into account the coordination between multiple muscles, providing a more accurate and dynamic real-time measure of fatigue, especially during coordinated multi-joint tasks.

This study extends our previous research by conducting comprehensive biomechanical modeling, quantitative muscle synergy-based rehabilitation assessment, and objective fatigue monitoring during piano-based upper limb rehabilitation. Specifically, this research aims to (1) establish a detailed biomechanical model (Hill-type muscle modeling) clarifying interactions among the piano keys, fingers, and exoskeleton; (2) implement muscle synergy analysis (using EMG signals and NMF) to quantitatively evaluate patients’ rehabilitation progress and neuromuscular coordination; and (3) introduce an innovative Comprehensive Muscle Fatigue Index (CMFI) model to objectively monitor fatigue, enhancing safety and optimizing therapeutic efficacy. By systematically addressing biomechanical, evaluative, and fatigue-monitoring gaps, this study significantly advances the therapeutic potential and clinical applicability of piano-integrated upper limb rehabilitation.

## 2. Materials and Methods

### 2.1. Construction of Biomechanical Models

To investigate the interactions and force characteristics among the fingers, piano keys, and exoskeleton during piano-based occupational therapy, this study established a comprehensive dynamic model of the “key–finger–exoskeleton” system based on the Hill-type muscle model and rigid-body dynamics theory. The model construction was divided into three stages: first, the development of the dynamic interaction model between the piano keys and the fingers; second, the development of the dynamic interaction model between the fingers and the exoskeleton; and finally, the integration of these sub-models into a complete biomechanical model.

#### 2.1.1. Dynamic Model Between Piano Key and Finger

During the process of finger pressing, the piano key exhibits typical elastic characteristics. To accurately describe this behavior, the key was modeled as a linear spring–damper system, and the feedback force generated by the key can be expressed as:

(1) $$F=k_{s}x_{k}+b_{s}{\dot{x}}_{k}$$

where F represents the elastic feedback force exerted by the key on the finger, $k_{s}$ denotes the stiffness coefficient of the piano key, $b_{s}$ denotes the damping coefficient, and $x_{k}$ and ${\dot{x}}_{k}$ represent the displacement and velocity of the key, respectively. These parameters were determined through experimental measurements of the force–displacement characteristics of the piano keys.

The finger was simplified as a multi-joint rigid linkage structure, with each finger consisting of multiple rigid phalanges. According to anatomical structures, the index, middle, ring, and little fingers were modeled with three phalanges each, while the thumb was modeled with two phalanges. The joint motions are driven by the combined effects of muscle contraction torques, key feedback torques, and exoskeleton assistance torques. The motion equation for the finger joints can be described as:

(2) $$M_{f}(\theta )\ddot{\theta }+C_{f}(\theta ,\dot{\theta })\dot{\theta }=\tau_{m}-\tau_{k}-\tau_{e}$$

where $M_{f}(\theta )$ is the inertia matrix of the finger joints, $C_{f}(\theta ,\dot{\theta })$ represents the Coriolis and centrifugal forces, $\tau_{m}$ is the joint torque generated by active muscle contraction, $\tau_{k}$ is the feedback torque generated by the piano key, and $\tau_{e}$ is the assistance torque provided by the exoskeleton. The feedback torque from the key acting on the fingertip is converted into joint torques via the Jacobian matrix, expressed as:

(3) $$\tau_{k}=J_{k}^{T}(\theta )F$$

where $J_{k}^{T}(\theta )$ is the Jacobian matrix relating the joint space of the finger to the fingertip contact point, obtained through kinematic analysis.

#### 2.1.2. Dynamic Model Between Finger and Exoskeleton

The primary function of the finger exoskeleton is to assist patients in completing rehabilitation movements. The exoskeleton is directly connected to each finger joint via a linkage structure. The driving force of the exoskeleton is generated by a linear push-rod actuator combined with a mechanical linkage transmission system, and its dynamics can be described by the following equation:

(4) $$M_{e}(\theta )\ddot{\theta }+C_{e}(\theta ,\dot{\theta })\dot{\theta }+K_{s}(\theta -\theta_{eq})=\tau_{e}$$

where $M_{e}(\theta )$ and $C_{e}(\theta ,\dot{\theta })$ represent the inertia and damping terms of the exoskeleton, respectively; $K_{s}$ is the (constant) structural stiffness matrix of the mechanism; and $\theta_{eq}$ is the equilibrium configuration imposed by gravity compensation.

Assuming rigid connections between the exoskeleton and the finger joints, the assistance torques generated by the exoskeleton are directly applied to the corresponding finger joints. The assistance torque is produced based on the deviation between the actual joint angles and the desired positions:

(5) $$\tau_{e}=K_{p}(\theta_{d}-\theta )+K_{d}({\dot{\theta }}_{d}-\dot{\theta })$$

where $K_{p}$ and $K_{d}$ are proportional and derivative gains, $\theta_{d}$ is the desired joint trajectory, and ${\dot{\theta }}_{d}$ its velocity.

The matrices $K_{s}$, $K_{p}$, and $K_{d}$ were obtained from combined experimental calibration and finite element analysis (FEA) to ensure accurate and reliable torque output.

#### 2.1.3. Overall Biomechanical Model of the Key–Finger–Exoskeleton System

Based on the two aforementioned sub-models, we established the overall dynamic equation for the interaction among the piano key, finger, and exoskeleton. By integrating the key feedback force, the exoskeleton assistance force, and the active muscle contraction torque, the complete biomechanical system equation is formulated as follows:

(6) $$\left[M_{f}(\theta )+M_{e}(\theta )]\ddot{\theta }+[C_{f}(\theta ,\dot{\theta })+C_{e}(\theta ,\dot{\theta })]\dot{\theta }+K_{e}(\theta -\theta_{d})=\tau_{m}-J_{k}^{T}(\theta )(k_{s}x_{k}+b_{s}{\dot{x}}_{k}\right)$$

This equation clearly describes the dynamic relationships among the finger joints during the piano-playing occupational therapy process, thus providing a theoretical foundation for subsequent simulation analysis of the system.

In this study, the active muscle contraction torque $\tau_{m}$ was modeled using the Hill-type muscle model. Its specific expression is given by:

(7) $$\tau_{m}=F^{\max}\cdot f({\tilde{l}}^{m}(t))\cdot f({\tilde{v}}^{m}(t))\cdot \alpha_{m}(t)\cdot r_{m}(t)$$

where $F^{\max}$ denotes the maximum isometric force of the muscle; $f({\tilde{l}}^{m}(t))$ represents the force–length relationship function between the muscle fiber length and the maximal muscle force coefficient; $f({\tilde{v}}^{m}(t))$ represents the force–velocity relationship function between the maximal muscle force coefficient and the muscle fiber contraction velocity; $\alpha_{m}(t)$ denotes the muscle activation level, also referred to as muscle activation degree or muscle activity level; and $r_{m}(t)$ refers to the moment arm of the muscle force acting on the joint. These parameters were determined based on anatomical data and existing literature, and were further calibrated and corrected using experimentally collected surface electromyography (sEMG) signals and motion capture data.

**Figure 1:**
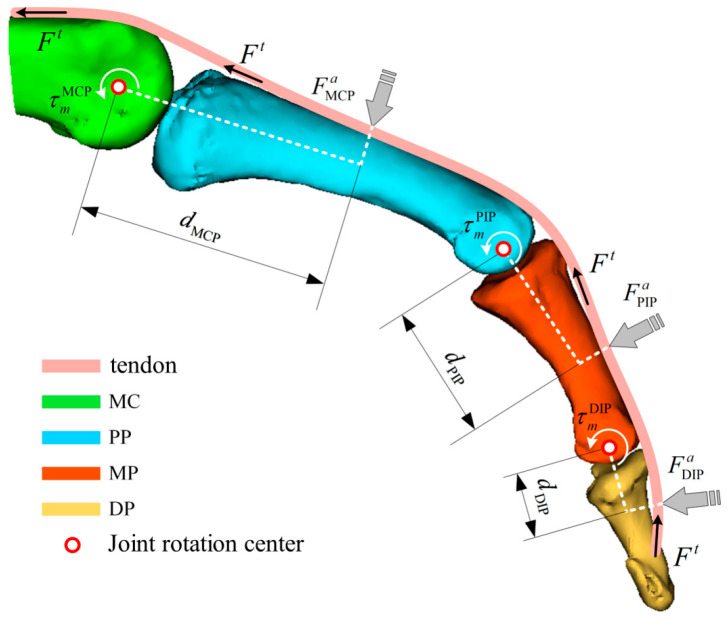
Force analysis of fingers based on a muscle bone model (MC: metacarpal; PP: proximal phalanx; MP: middle phalanx; DP: distal phalanx).

By combining the muscle contraction dynamics model and the muscle activation dynamics model, the resultant force $F^{t}$ acting on the joints can be solved. However, $F^{t}$ represents the overall tendon force on a single finger. Similar to the driving principle of tendon-driven hand rehabilitation robots, $F^{a}$ is uniformly distributed across each joint of a single finger and thus cannot directly determine the desired assistive force F that the hand rehabilitation robot should apply to the finger. Therefore, $F^{t}$ cannot be directly used for single-joint endpoint force control in hand rehabilitation robotics. To address this, it is necessary to further compute the joint torques $\tau_{m}^{\mathrm{MCP}}(t)$, $\tau_{m}^{\mathrm{PIP}}(t)$, and $\tau_{m}^{\mathrm{DIP}}(t)$ generated by F’ at each finger joint. Subsequently, by incorporating the distances from the robot’s force application points to the instantaneous centers of rotation of the corresponding joints, namely $d_{\mathrm{MCP}}$, $d_{\mathrm{PIP}}$, and $d_{\mathrm{DIP}}$, the desired force $F^{a}$ required for force control in the hand rehabilitation robot can be calculated, as illustrated in the Figure 1.

To further compute the joint torques, it is necessary to perform a geometrical analysis of the musculoskeletal model to determine the corresponding moment arms $r_{m}(t)$ at the joint locations. The moment arm $r_{m}(t)$ is a function that varies with both the muscle length and the joint angle. According to the principle of virtual work, the following relationship can be derived:

(8) $$\tau_{m}(t)\Delta \theta_{m}(t)=F^{t}(t)\Delta l^{mt}(t)$$

where $\tau_{m}(t)$ represents the joint torque, $\Delta \theta_{m}(t)$ is the virtual angular displacement, $F^{t}(t)$ is the muscle force, and $\Delta l^{mt}(t)$ is the virtual change in the total muscle tendon length, which depends on the muscle tendon unit length $l^{t}(t)$ and the joint angle $\theta_{m}$.From this relationship, the moment arm $r_{m}(t)$ can be expressed as:

(9) $$r_{m}(t)=\frac{\partial l^{mt}(\theta_{m})}{\partial \theta_{m}}$$

Subsequently, the joint torque generated at a single joint of the finger can be expressed as:

(10) $$\tau_{m}(t)=F^{t}(t)\cdot r_{m}(t)$$

Finally, based on the above derivations, the expected assistive force $F^{a}(t)$ applied by the hand rehabilitation robot to each finger segment can be obtained.

(11) $$F^{a}(t)=\frac{\tau_{m}(t)}{d_{\mathrm{joint}}(t)}$$

where $d_{\mathrm{joint}}(t)$ denotes the distance between the force application point of the hand rehabilitation robot and the instantaneous center of rotation of the corresponding joint.

### 2.2. Muscle Synergy-Based Rehabilitation Assessment Method

The muscle synergy theory hypothesizes that during the execution of specific movements, different muscles are activated in specific patterns of coordination. To further investigate the rehabilitation effectiveness of the piano-based occupational therapy device, this study employed surface electromyography (sEMG) technology and proposed a muscle coordination analysis method based on non-negative tensor factorization (NTF) to quantitatively assess changes in patients’ neuromuscular coordination during rehabilitation.

#### 2.2.1. Construction of the sEMG Tensor

Muscle synergy analysis is commonly performed using non-negative matrix factorization (NMF). Originally applied in image processing, NMF has been widely adopted in physiological signal analysis due to its non-negativity, interpretability, and sparsity properties. It has been extensively used to reveal intermuscular coordination relationships from multichannel EMG signals by decomposing the original matrix into two lower-dimensional subspaces. Compared with traditional two-dimensional muscle synergy analysis, three-dimensional synergy analysis can capture more comprehensive features of EMG signals, making it more suitable for analyzing hand motion patterns. Based on NMF for two-dimensional decomposition, this study extends the model to non-negative tensor factorization (NTF) for decomposing three-dimensional EMG tensors, which incorporates frequency, time, and space (frequency × time × space). This approach allows for a more comprehensive assessment of muscle coordination by considering the temporal, spatial, and frequency domain features of muscle activation. By including the frequency dimension, our method captures subtle variations in muscle activity that are particularly relevant in piano rehabilitation, where rapid muscle shifts are required for key presses. This 3D synergy analysis offers a more accurate and nuanced understanding of muscle synergies, making it particularly suited for tasks involving fine motor control and dynamic muscle activation patterns, such as piano-based rehabilitation.

First, it is necessary to construct the EMG tensor. In the NMF model, the EMG matrix $E_{n_{e}\times t}$ is composed of time-series EMG signals e(t) recorded across $n_{e}$ channels, which can be directly obtained through data acquisition. However, in the NTF model, the EMG tensor cannot be directly acquired from measurements. To construct the EMG tensor, it is essential to first define the physical meaning of each dimension. Based on the two dimensions of the EMG matrix, an additional frequency domain is introduced, resulting in a three-dimensional EMG tensor with the following structure: frequency domain f (frequency features of e(t)) × time domain t (time-series EMG data e(t)) × spatial domain s (number of channels), denoted as $E_{f\times t\times s}\in R_{+}^{f\times t\times s}$.

The frequency dimension f of the EMG tensor is obtained through continuous wavelet transform (CWT). Since the main energy components of EMG signals are concentrated within the range of 0 to 150 Hz, the Morlet wavelet is selected as the mother wavelet for the transformation:

(12) $$\psi (t)=\frac{1}{\sqrt{\sigma }\pi^{1/4}}e^{j\omega_{0}t}e^{-\frac{t^{2}}{2\sigma^{2}}}$$

where ψ(t) represents the Morlet wavelet function, σ is the standard deviation of the wavelet function, and $\omega_{0}$ is the center frequency of the wavelet function. Continuous wavelet decomposition is performed using the Morlet wavelet function, and the continuous wavelet coefficients are obtained as follows:

(13) $$C_{i}=\int_{-\infty }^{\infty }e(t)\cdot \psi (t-t_{i})dt$$

where $C_{i}$ represents the continuous wavelet coefficient at time point $t_{i}$. After traversing all the time-series data points of e(t), the column vectors $C_{1},C_{2},\ldots ,C_{t}$ are combined to construct a matrix $C_{f\times t}$:

(14) $$C_{f\times t}=[C_{1};C_{2};\ldots ;C_{t}]$$

where f denotes the frequency scale parameter set in the wavelet analysis. The relationship between the frequency scale parameter f and the Morlet wavelet parameters is given by:

(15) $$f=\frac{f_{c}\cdot F_{s}}{s_{c}}$$

where $f_{c}$ is the center frequency of the Morlet wavelet, typically set to approximately 0.849; $F_{s}$ is the sampling rate of the EMG signal (downsampled to 400 Hz); and $s_{c}$ is the scale parameter, set to 30. The continuous wavelet coefficient matrix $C_{f\times t}(f,t)$ simultaneously captures both the time domain and frequency domain features of a single-channel EMG signal. Each matrix slice $C_{f\times t}(f,t)$ corresponds to one channel. Assuming that s channels are recorded, the complete EMG tensor is constructed by stacking s such $C_{f\times t}(f,t)$ matrices along the spatial dimension.

(16) $$E_{f\times t\times s}(f,t,j)=C_{f\times t}^{\left(j\right)}(f,t),\;j=1,2,\ldots ,s$$

#### 2.2.2. Non-Negative Tensor Factorization Based on CP-ALS Algorithm

After constructing the EMG tensor, it is necessary to perform non-negative tensor factorization to extract the muscle synergy characteristics across channels. Common tensor decomposition methods include principal component analysis (PCA), CP (Candecomp/Parafac) decomposition [24], and Tucker decomposition. PCA is suitable for highly correlated data and can retain the principal components of the original information. CP decomposition factorizes a three-dimensional tensor into the outer products of multiple matrices, with each matrix representing a feature in one mode. Tucker decomposition, suitable for higher-order tensors, factorizes the tensor into a core tensor and multiple mode matrices.

Considering the characteristics of EMG signals and the requirements of muscle system analysis, CP decomposition based on the alternating least squares (ALS) algorithm was adopted for non-negative tensor factorization. Initially, the factor matrices were initialized, and the rank $r_{d}$ (i.e., the number of muscle synergies) was assigned based on prior knowledge. Subsequently, the value of $r_{d}$ was adjusted according to the reconstruction accuracy.

(17) $$E_{f\times t\times s}\approx \sum_{r=1}^{r_{d}}F_{r}\circ T_{r}\circ S_{r}$$

The factor matrices $F_{r}$, $T_{r}$, and $S_{r}$ must satisfy $F_{r}=[f_{1},f_{2},\ldots ,f_{r}]\in {R_{+}}^{f\times R}$, representing the frequency coefficient matrix; $T_{r}=[t_{1},t_{2},\ldots ,t_{r}]\in {R_{+}}^{t\times R}$, representing the activation scaling coefficient matrix; and $S_{r}=[s_{1},s_{2},\ldots ,s_{r}]\in {R_{+}}^{s\times R}$, representing the muscle weight coefficient matrix.

The objective of CP decomposition is to find the optimal set of factor matrices {F, T, S} that minimize the approximation error, optimized using the ALS algorithm. The optimization is performed iteratively: Fix $F_{r}$ and $T_{r}$, update $S_{r}$ (as in Equation (18)), fix $S_{r}$ and $T_{r}$, update $F_{r}$ (as in Equation (19)), fix $F_{r}$ and $S_{r}$, and update $T_{r}$ (as in Equation (20)).

(18) $$S_{r}={\left(T_{r}^{T}T_{r}\circ F_{r}^{T}F_{r}\right)}^{-1}T_{r}^{T}E_{\left(3\right)}F_{r}$$

(19) $$F_{r}={\left(S_{r}^{T}S_{r}\circ T_{r}^{T}T_{r}\right)}^{-1}S_{r}^{T}E_{\left(1\right)}T_{r}$$

(20) $$T_{r}={\left(F_{r}^{T}F_{r}\circ S_{r}^{T}S_{r}\right)}^{-1}F_{r}^{T}E_{\left(2\right)}S_{r}$$

where $E_{\left(1\right)}$, $E_{\left(2\right)}$, and $E_{\left(3\right)}$ represent the matrix unfoldings of the EMG tensor $E_{f\times t\times s}$ along the frequency dimension, time dimension, and spatial dimension, respectively. The final goal is to find the optimal set of factor matrices $\left\{F_{r},T_{r},S_{r}\right\}$ such that the reconstructed tensor approximates the original tensor.

(21) $$\underset{\left\{F_{r},T_{r},S_{r}\right\}}{\min}\sum_{1}^{f}\sum_{1}^{t}\sum_{1}^{s}{\left(E_{f\times t\times s}-\sum_{r=1}^{R}F_{fr}T_{tr}S_{sr}\right)}^{2}$$

After the tensor decomposition, the three factor matrices are multiplied to reconstruct the data tensor, and the fitting value FIT is calculated to evaluate the reconstruction accuracy. The value of FIT ranges from 0 to 1, with a higher value indicating better reconstruction quality. The definition of FIT is as follows:

(22) $$FIT=1-\frac{\sqrt{{\left(\left\|X_{t}\right\|\right)}^{2}-{\left(\left\|Y_{t}\right\|\right)}^{2}}}{\left\|X_{t}\right\|}$$

where $X_{t}$ represents the original constructed tensor, and $Y_{t}$ represents the reconstructed data tensor obtained from the NTF algorithm. Following the convention in NMF-based muscle synergy decomposition, the optimal number of synergies is determined when the average FIT reaches or exceeds 90%, and the incremental improvement after increasing one synergy component is less than or equal to 2%.

#### 2.2.3. Rehabilitation Evaluation Based on Three-Dimensional Muscle Synergy Information

After extracting patients’ muscle synergy information, the spatial, temporal, and frequency domain components were compared against healthy subjects to assess rehabilitation progress. The Pearson correlation coefficient r was calculated to quantify the similarity between the synergy matrices of patients and healthy subjects:

(23) $$r=\frac{n\sum_{i}^{n}x_{i}y_{i}-\sum_{i}^{n}x_{i}\sum_{i}^{n}x_{i}}{\sqrt{n\sum_{i}^{n}x_{i}^{2}-{\left(\sum_{i}^{n}x_{i}\right)}^{2}}\sqrt{n\sum_{i}^{n}y_{i}^{2}-{\left(\sum_{i}^{n}y_{i}\right)}^{2}}}$$

where $x_{i}$ and $y_{i}$ are the compared datasets, n is the data length, and r is the correlation coefficient.

### 2.3. Muscle Fatigue Evaluation Based on Muscle Synergy Analysis

Currently, muscle fatigue evaluation primarily focuses on single-muscle strength assessments, with limited attention to fatigue under multi-muscle coordinated motion conditions. To address this, we propose a Comprehensive Muscle Fatigue Index (CMFI) model based on sEMG signals, combined with non-negative matrix factorization (NMF) to analyze the duration multi-muscle weight coefficient matrix (DMWCM). By extracting representative time domain, frequency domain, and complexity features, we quantitatively assess the fatigue level during coordinated multi-muscle movements.

#### 2.3.1. Construction of the Multi-Muscle Weight Coefficient Matrix Based on NMF

An EMG matrix $E_{n_{e}\times n_{s}}$ was constructed, where $n_{e}$ represents the total number of channels, meaning that EMG signals were collected from $n_{e}$ muscles. $n_{s}$ denotes the total number of sample points collected for each channel. Then, $E_{n_{e}\times n_{s}}$ was subjected to NMF decomposition. For example, if a three-channel EMG signal is decomposed into two synergy modes using the NMF algorithm, and at a certain moment the activation scaling coefficient of synergy mode 1 is greater than that of synergy mode 2, then synergy mode 1 is considered the dominant motor mode at that time. Conversely, if synergy mode 2 has a higher activation coefficient, it becomes the dominant mode. For the EMG matrix $E_{n_{e}\times n_{s}}$, assuming the number of synergy modes $r_{d}$ = 2, the dominant motor mode at any time t can be determined as:

(24) $$M_{all}={\left(\begin{array}{c} {\left[bool(T_{11}>T_{21}),bool(T_{11}<T_{21})\right]}^{T}\\ \vdots \\ {\left[bool(T_{1n_{s}}>T_{2n_{s}}),bool(T_{1n_{s}}<T_{2n_{s}})\right]}^{T} \end{array}\right)}^{T}$$

(25) $$M_{t}={\left[bool(T_{1t}>T_{2t}),bool(T_{1t}<T_{2t})\right]}^{T}$$

where $M_{all}$ represents the dominant motor mode at any given time, $M_{t}$ represents the dominant motor mode corresponding to time *t*, $T_{2n_{s}}$ represents the activation scaling coefficient of the $n_{s}$-th sample under the second synergy mode, and “*bool*” denotes performing a Boolean operation on the value inside the parentheses. Further, the corresponding DMWCM (duration multi-muscle weight coefficient matrix) can be calculated as:

(26) $$S^{main}=\left(\begin{array}{ccc} (S_{11}(1),\;S_{12}(1))M_{1} & \ldots & (S_{11}(n_{s}),\;S_{12}(n_{s}))M_{n_{s}}\\ \vdots & \ddots & \vdots \\ (S_{n_{e}1}(1),\;S_{n_{e}2}(1))M_{1} & \ldots & (S_{n_{e}1}(n_{s}),\;S_{n_{e}2}(n_{s}))M_{n_{s}} \end{array}\right)$$

(27) $$S^{main}(t)=\left(\begin{array}{c} \left(S_{11}(t),\;S_{12}(t))(M_{t}\right)\\ \vdots \\ \left(S_{n_{e}1}(t),\;S_{n_{e}2}(t))(M_{t}\right) \end{array}\right)$$

(28) $$s_{i}^{main}(t)=(S_{i1}(t),\;S_{i2}(t))(M_{t})$$

where $S^{main}$ represents the DMWCM (duration multi-muscle weight coefficient matrix). $S_{n_{e}2}(n_{s})$ refers to the muscle weight coefficient under the activation scaling coefficient of the $n_{s}$-th sample at the $n_{e}$-th channel in the second synergy mode. In simpler terms, it is the muscle weight coefficient of the $n_{e}$-th channel corresponding to $T_{2n_{s}}$. $M_{n_{s}}$ represents the dominant motor mode corresponding to the $n_{s}$-th sample. $M_{n_{s}}$ can also be expressed in terms of time. If the sampling frequency is $f_{e}$, then $M_{n_{s}}=M_{n_{s}/f_{e}}=M_{t_{s}}$, where $t_{s}$ is the sampling period, and the expressions of $M_{n_{s}}$ and $M_{t_{s}}$ are consistent. $s_{i}^{main}(t)$ represents the muscle weight coefficient of the i-th muscle at time *t* under the dominant motor mode.

#### 2.3.2. Construction of the CMFI Model Based on DMWCM and Multi-Feature Fatigue Indicators

Fatigue features refer to the statistical and morphological characteristics extracted from the time series of EMG signals. Information from aspects such as signal amplitude, waveform, and distribution is considered. Specifically, four EMG fatigue feature parameters are used: root mean square (RMS), median frequency (MF), permutation entropy (PE), and fractal dimension (FD). These parameters are integrated to obtain the fatigue index of a single muscle:

(29) $$I_{i}^{\mathrm{FMI}}=T_{R}\cdot r_{fi}+T_{M}\cdot m_{fi}+T_{P}\cdot p_{fi}+T_{F}\cdot f_{fi}$$

where $r_{f}$, $m_{f}$, $p_{f}$, and $f_{f}$ represent the normalized values of RMS, MF, PE, and FD, respectively. $I_{i}^{\mathrm{FMI}}$ denotes the fatigue index value of each muscle. $T_{R}$, $T_{M}$, $T_{P}$, and $T_{F}$ are the corresponding weighting coefficients for each fatigue feature.

By combining the DMWCM matrix obtained through NMF, the CMFI value under multi-muscle coordinated motion can be calculated as:

(30) $$I^{\mathrm{CFMI}}=\sum_{i=1}^{n_{e}}s_{i}^{main}(t)\cdot I_{i}^{\mathrm{FMI}}$$

where $I^{\mathrm{CFMI}}$ represents the CMFI value under multi-muscle coordinated motion, and i denotes the i-th muscle.

## 3. Experiment and Result Analysis

To validate the effectiveness and applicability of the proposed biomechanical model, rehabilitation assessment method, and fatigue-monitoring method, three experiments were designed and conducted: a biomechanical model validation experiment, a rehabilitation effectiveness assessment experiment, and a fatigue-monitoring validation experiment. Three healthy subjects and three stroke patients (average age 55 ± 8 years) participated. The sample size for this study was chosen based on the experimental design and available resources. Three stroke patients (average age 55 ± 8 years) were selected according to the following inclusion criteria: (1) upper-limb motor impairment, (2) no significant cognitive impairment, and (3) no severe comorbidities. The primary purpose of this study was to validate the biomechanical model, muscle synergy-based assessment, and fatigue-monitoring methods. Although a larger sample size is desirable for more robust statistical analysis, the three patients in this study provide preliminary insights into the feasibility and potential of the proposed methods. Future research will include a larger participant pool and conduct power analysis to determine an adequate sample size. The experimental protocol was approved by the Ethics Committee of Chengde Medical University (approval number: 2025002), and informed consent was obtained from all subjects involved in the study.

### 3.1. Biomechanical Model Validation Experiment

The primary objective of this experiment was to verify the accuracy and validity of the established “key–finger–exoskeleton” biomechanical model, with particular focus on the precision of the predicted interaction torques between the piano key and the finger, and between the finger and the exoskeleton.

The experimental task involved patients continuously pressing piano keys to complete a designated simple melody fragment. Each patient repeated the task three times to ensure data stability and reliability. The collected mechanical and kinematic data were used for comparative validation against the simulation data generated by the biomechanical model.

A multi-node pressure sensing system was designed to collect all interaction forces between the “key–finger–exoskeleton.” The sensing system employed the MM32spin27ps microcontroller as the main control chip, featuring a 12-bit ADC with a sampling speed of up to 1 MHz. In order to capture the complete hand interaction and contact forces, at least 14 force-sensing nodes were required. Therefore, the system utilized a 16-channel ADC composed of ADC1 and ADC2 (with two channels left unused) to perform real-time pressure data acquisition at a sampling frequency of 200 Hz.

**Figure 2:**
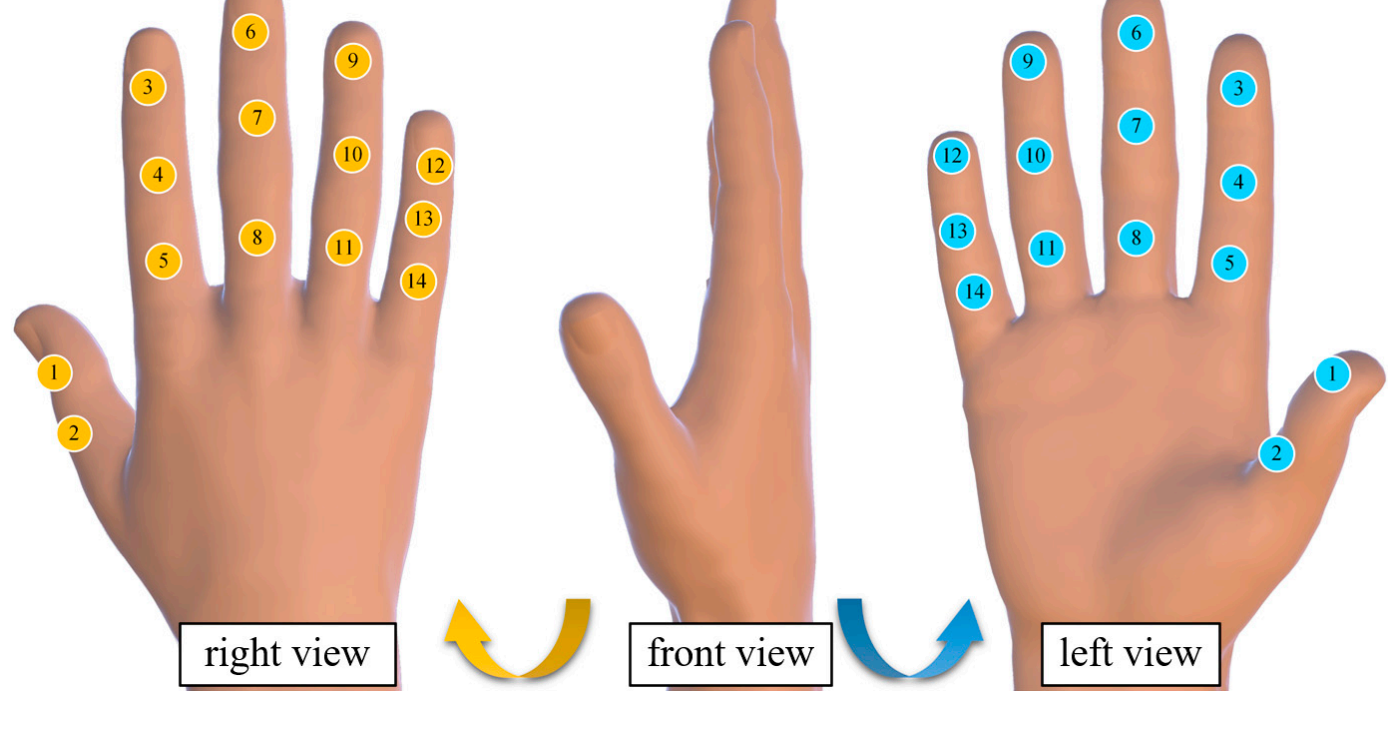
Hand force perception system node distribution diagram. (The yellow icon represents the sensor pasted on the back of the hand, the blue icon represents the sensor on the palm, and the numbers represent the sensor number.)

Two main control circuits were deployed to achieve synchronized acquisition of interaction forces and contact forces, resulting in a total of 28 force-sensing nodes, thus constructing a complete hand force-sensing system. The selected thin-film pressure sensors were the model C5-ST-LF5, with a measurement range of 5–600 g, a resolution of 5 g (approximately 0.05 N), and a response time of less than 0.01 ms. The sensors are compact and highly sensitive, allowing them to be fully attached to various fingertip positions for real-time pressure data collection. The distribution of interaction force and contact force sensing nodes is shown in Figure 2.

**Figure 3:**
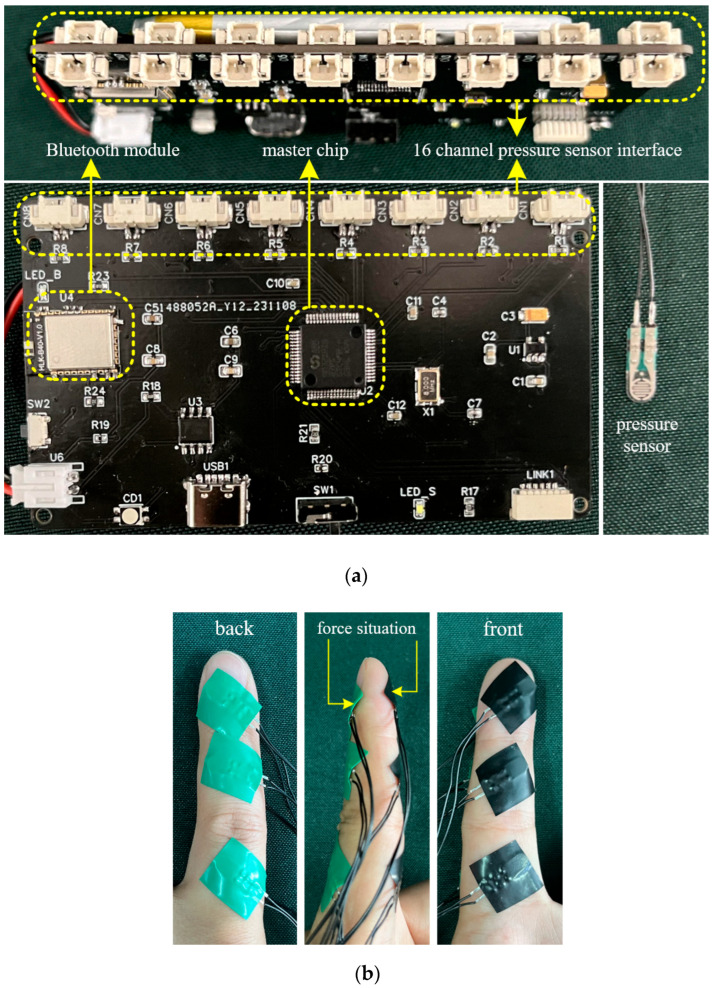
Multi-node hand force perception system (**a**) and pressure sensor attachment method (**b**).

Due to the small adhesive area of the built-in sensor stickers, the sensors were prone to detachment after sticking; therefore, additional fixation was performed using insulating tape. The detailed structure of the multi-node force-sensing system and the method of pressure sensor attachment are shown in Figure 3.

**Figure 4:**
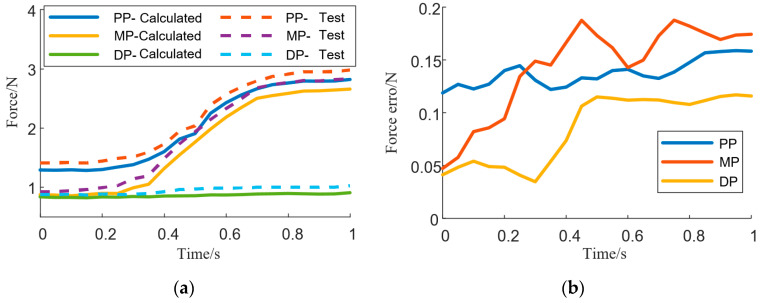
Comparison between the calculated force of fingers and the actual force applied (**a**), and the difference between the two forces (**b**) (PP: proximal phalanx; MP: middle phalanx; DP: distal phalanx).

The force data associated with each finger during exoskeleton-assisted key pressing were processed by extracting the rising edge (i.e., the execution force data during the transition from resting state to initial contact with the object) and the falling edge (i.e., the force data during the motion process from detachment from the object back to resting state). For each finger, the average values of all rising edges and falling edges were calculated separately. Subsequently, a comparative analysis was conducted between the computed forces and the measured interaction forces for different finger segments, focusing on the rising edge data. The error between the measured interaction force and the biomechanical model-calculated force was then computed. Taking the index finger as an example, the comparison is shown in Figure 4. The error data for all fingers are summarized in Table 1.

### 3.2. Rehabilitation Effectiveness Assessment Experiment

The objective of this experiment was to verify the accuracy, sensitivity, and clinical applicability of the rehabilitation effectiveness assessment method based on muscle synergy analysis proposed in this study. During the experiment, surface EMG signals were collected from patients performing piano-playing occupational therapy tasks. The collected signals were used to construct EMG tensors, and muscle coordination analysis was conducted. A similarity analysis was performed between the patients’ three-dimensional muscle coordination information and that of healthy subjects performing the same tasks, using the correlation coefficient as the evaluation metric. In parallel, traditional rehabilitation assessments, including the Fugl–Meyer Assessment and the Box-and-Block Test, were conducted as reference standards.

**Figure 5:**
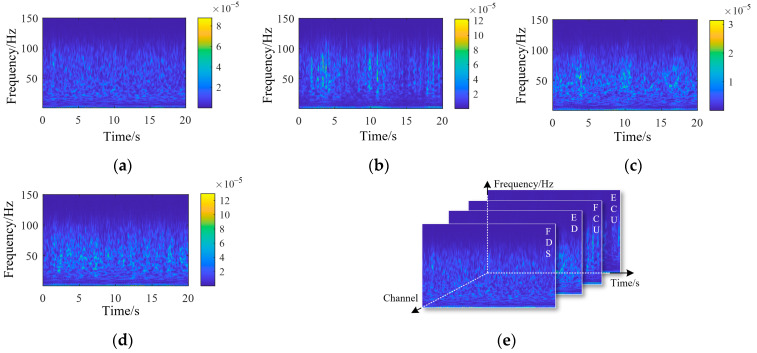
Wavelet continuous decomposition coefficients of EMG signals from each channel: (**a**) FDS, (**b**) ED, (**c**) FCU, (**d**) ECU, and (**e**) the composition of the EMG tensor. (ED: extensor digitorum; FDS: flexor digitorum superficialis; ECU: extensor carpi ulnaris; FCU: flexor carpi ulnaris).

Surface EMG signals were collected from the extensor digitorum (ED), flexor digitorum superficialis (FDS), extensor carpi ulnaris (ECU), and flexor carpi ulnaris (FCU). The continuous wavelet transforms coefficients of each muscle, together with the constructed EMG tensor, are shown in Figure 5.

**Figure 6:**
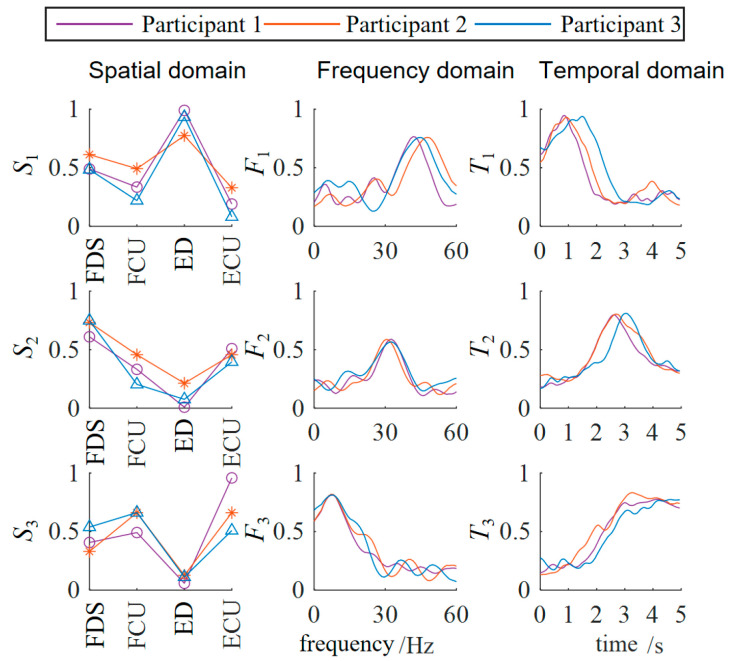
Three-dimensional muscle synergy analysis of healthy subjects. (ED: extensor digitorum; FDS: flexor digitorum superficialis; ECU: extensor carpi ulnaris; FCU: flexor carpi ulnaris).

The three-dimensional muscle synergy information of the healthy subjects is shown in Figure 6.

**Figure 7:**
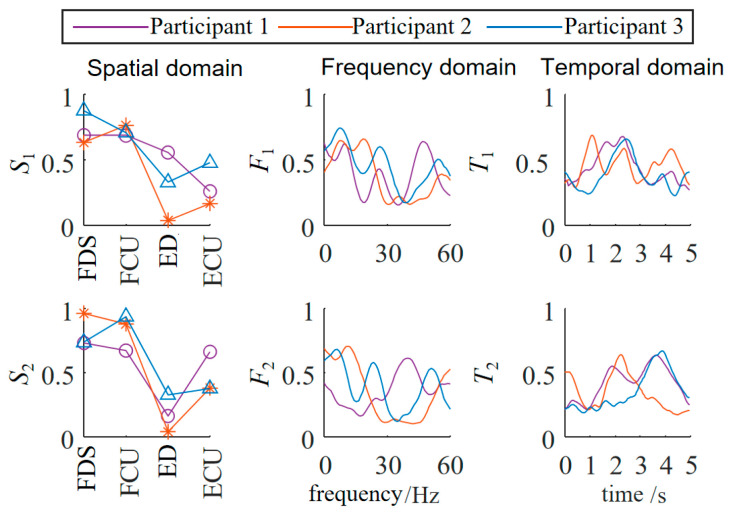
Three-dimensional muscle synergy analysis of patients (first occupational therapy).

The three-dimensional muscle synergy information of the patients during their initial session of occupational therapy is shown in Figure 7.

**Figure 8:**
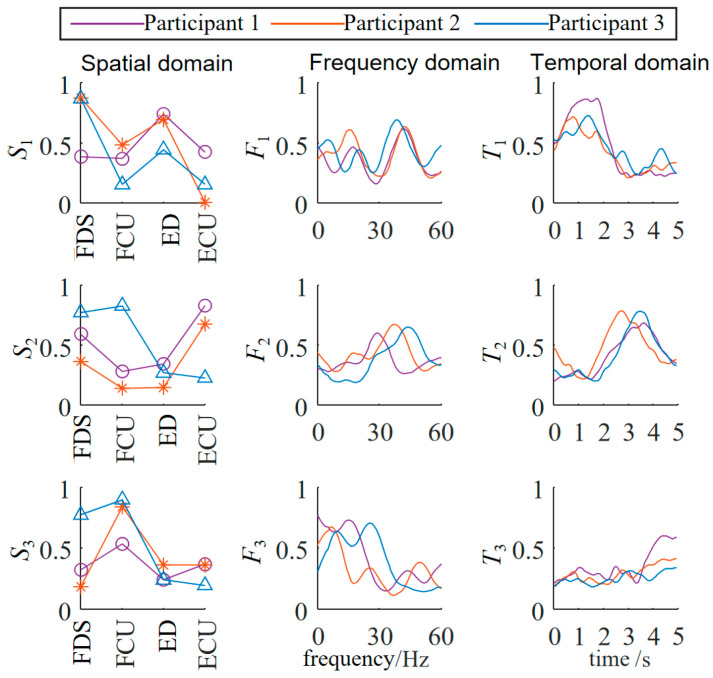
Three-dimensional muscle synergy analysis of patients (three weeks of continuous occupational therapy).

The three-dimensional muscle synergy information of the patients after three weeks of continuous occupational therapy is shown in Figure 8.

Through data analysis, it was found that after three weeks of occupational therapy, all three patient subjects exhibited an increase of one additional synergy mode. The S1 similarities increased to 0.8502, 0.7071, and 0.4632, while the S2 similarities increased to 0.5714, 0.5151, and 0.4747. The newly added S3 synergy mode also showed positive correlations with the S3 synergy mode of the healthy subjects, with Patient 1 demonstrating a strong positive correlation.

The F1 similarities increased to 0.5948, 0.3569, and 0.4231, while the F2 similarities increased to 0.8140, 0.6374, and 0.1317. The newly added F3 synergy mode also showed a positive correlation with the third synergy mode of the healthy subjects. The T1 similarities increased to 0.9122, 0.9540, and 0.8863, while the T2 similarities increased to 0.7582, 0.7381, and 0.6600. The newly added T3 synergy mode also showed a strong positive correlation with that of the healthy subjects. The temporal domain muscle synergies of the patient subjects reached a relatively high level, overall about twice as high as the similarities in the spatial and frequency domains, indicating that the patients’ EMG temporal activation patterns were significantly optimized and tended toward normal patterns.

This result from the muscle coordination analysis was also consistent with the trends observed using traditional rehabilitation indicators, supporting the validity of rehabilitation assessment based on muscle coordination analysis.

### 3.3. Fatigue-Monitoring Validation Experiment

The objective of this experiment was to evaluate the effectiveness and real-time applicability of the Comprehensive Muscle Fatigue Index (CMFI) proposed in this study under actual training conditions, and to verify its clinical effectiveness in preventing patient overfatigue.

**Figure 9:**
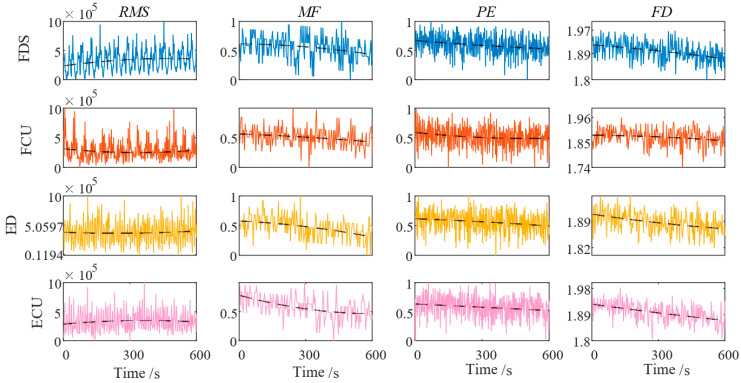
Fatigue characteristics of muscles in each channel. (RMS: root mean square; MF: median frequency; PE: permutation entropy; FD: fractal dimension).

Each patient performed a continuous 3 min piano-playing rehabilitation task, during which the task difficulty was gradually increased to induce muscle fatigue. During the experiment, the CMFI was calculated in real time. When the CMFI value reached the preset threshold, the system automatically prompted the patient to pause the training for rest, and training was resumed only after the CMFI value dropped below the threshold. The fatigue feature data of patients during the occupational therapy process are shown in Figure 9.

**Figure 10:**
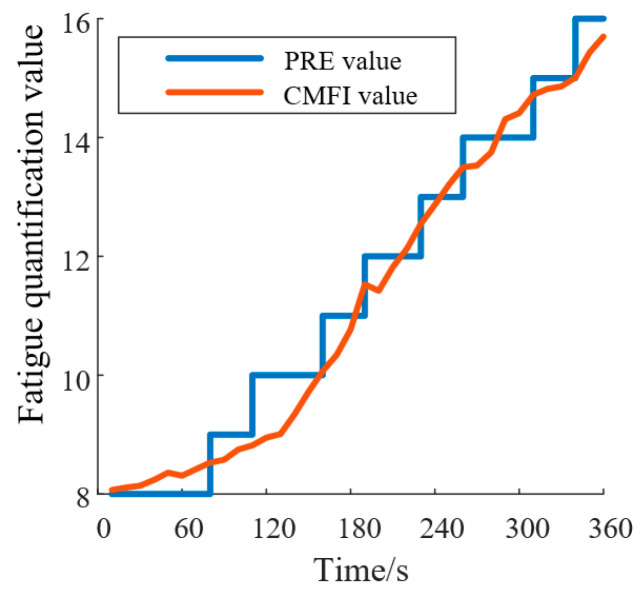
CMFI and PRE fitting results. (PRE: perceived rate of exertion; CMFI: Comprehensive Muscle Fatigue Index).

Simultaneously, during the experiment, researchers recorded the patients’ perceived fatigue levels using the perceived rate of exertion (PRE) scale as a subjective reference. After the experiment, statistical analysis was conducted to evaluate the correlation between the CMFI and the subjective fatigue scores, assessing the validity and real-time performance of the CMFI fatigue-monitoring method. Pearson correlation analysis and linear regression analysis were used to determine the predictive ability of the CMFI for subjective fatigue perception, as illustrated in Figure 10.

By calculating the Pearson correlation coefficients between the CFMI and the PRE curves for all subjects, a significant Pearson correlation was found between the CFMI and PRE fatigue curves (r > 0.83, *p* < 0.001). This result indicates that the relationship between the CFMI and PRE fatigue curves is statistically significant and practically meaningful. The CFMI can therefore be used as an indicator for fatigue monitoring during rehabilitation training, providing a more objective evaluation of fatigue levels based on EMG signals compared to the subjective PRE scale.

## 4. Discussion

In this study, we established a comprehensive biomechanical model of the “key–finger–exoskeleton” system, developed a three-dimensional muscle synergy-based rehabilitation assessment method, and proposed the real-time Comprehensive Muscle Fatigue Index (CMFI) for fatigue monitoring during piano-based occupational therapy tasks.

The biomechanical model was validated through multi-node pressure-sensing experiments. The results demonstrated that the predicted interaction forces between the piano key, the finger, and the exoskeleton closely matched the measured forces, with acceptable errors across different finger segments. This confirms that the proposed model accurately reflects the dynamic characteristics of the hand during piano-playing rehabilitation, providing a solid foundation for subsequent control and optimization of the rehabilitation device.

While the biomechanical model demonstrated promising results, some discrepancies in force predictions were observed, particularly for the little finger, where the prediction error reached 25.98%. These errors can be attributed to several factors: (1) sensor limitations: The pressure sensors used in this study have limited sensitivity and resolution, especially at lower force levels. The little finger, with its smaller muscle mass and weaker force production, is more prone to measurement errors, particularly at the distal phalanx joint, where forces are relatively low. (2) Anatomical variability: The little finger is anatomically distinct from the other fingers, with smaller muscles and joints that may vary significantly between individuals. Differences in tendon length, muscle fiber composition, and joint angles can contribute to larger prediction errors, especially at the distal joints (DP), where force interactions are smaller. (3) Model assumptions: The biomechanical model assumes rigid body mechanics and simplifies joint dynamics, which may not fully capture the complex interactions in the smaller joints of the little finger. This simplification leads to higher prediction errors, particularly in joints with lower force production and more intricate kinematic behavior. To quantify the impact of these factors, we performed a sensitivity analysis, which showed that variations in joint stiffness and muscle activation levels had the most significant influence on the predicted forces. The little finger joints, where the interaction forces are smaller, showed higher sensitivity to these changes, leading to larger discrepancies in force predictions. In addition, by combining the muscle contraction dynamics model and the muscle activation dynamics model, the resultant force $F^{t}$ acting on the joints can be solved. However, $F^{t}$ represents the overall tendon force on a single finger. Similar to the driving principle of tendon-driven hand rehabilitation robots, $F^{a}$ is uniformly distributed across each joint of a single finger and thus cannot directly determine the desired assistive force F that the hand rehabilitation robot should apply to the finger.

The muscle synergy-based rehabilitation assessment was also verified. In this study, both NMF and NTF showed excellent reconstruction accuracy, with FIT values above 95% and only a minimal difference in performance [24]. While NTF provided a more detailed analysis by considering the frequency domain, the added complexity did not result in a lower FIT value, making NTF a justified choice for more comprehensive muscle synergy analysis. After three weeks of occupational therapy, all patient subjects showed an increase in the number of synergy modes, and the similarities of spatial, temporal, and frequency domain synergy structures with healthy subjects significantly improved. In particular, the improvements in the temporal domain were approximately twice those in the spatial and frequency domains, suggesting that temporal muscle activation patterns were significantly optimized. This indicates that the three-dimensional synergy analysis method is highly sensitive in capturing rehabilitation progress, consistent with improvements observed in traditional clinical assessment scales such as the Fugl–Meyer Assessment and the Box-and-Block Test.

Furthermore, the CMFI fatigue-monitoring method was validated in real-time rehabilitation scenarios. The weighting coefficients used in the CMFI were initially selected based on prior studies in muscle fatigue analysis. However, to empirically validate these weights, we conducted a regression analysis using real-time fatigue data obtained from both subjective fatigue scores (e.g., perceived rate of exertion (PRE) scale) and objective physiological measures of fatigue (e.g., EMG amplitude, frequency shifts). The goal was to optimize the weights such that the CMFI provides the best fit to the ground truth fatigue measures. We employed a least squares regression approach to minimize the prediction error between the CMFI and the ground truth fatigue data. The final weights were determined based on the analysis, resulting in a correlation of r>0.83 between the CMFI and subjective fatigue ratings, confirming the validity of the weighting scheme. The results show that the CMFI exhibited a strong Pearson correlation (r > 0.83, *p* < 0.001) with subjective fatigue scores measured by the PRE scale. This confirms that the CMFI can objectively and accurately monitor fatigue levels based on EMG signals, providing a more real-time and quantifiable alternative to subjective fatigue ratings. The fatigue-monitoring mechanism, which automatically adjusted training intensity based on CMFI thresholds, effectively prevented overfatigue during therapy sessions.

However, the conclusions drawn from this study are limited by the small sample size (n = 3). Although the results are promising and align with findings from similar studies [25,26], the small sample size restricts the generalizability of these results. Previous research on task-oriented rehabilitation therapies, such as piano-based rehabilitation, has emphasized the importance of larger sample sizes to provide more robust evidence [27]. Therefore, while our study provides preliminary insights, further research with a larger participant pool is needed to validate these findings and refine the rehabilitation methods.

In conclusion, the combination of biomechanical modeling, muscle synergy-based evaluation, and real-time fatigue monitoring offers a promising framework for optimizing rehabilitation therapy, particularly for stroke patients and individuals with upper-limb impairments. These methods could potentially improve the personalization and safety of rehabilitation, but further studies with larger sample sizes are required to confirm the efficacy of these approaches.

## 5. Conclusions

This study presented a comprehensive framework for piano-based upper-limb rehabilitation, integrating biomechanical modeling, three-dimensional muscle synergy analysis, and real-time fatigue monitoring. The established “key–finger–exoskeleton” biomechanical model accurately described the dynamic interactions during piano-playing tasks and was validated through force measurement experiments. The three-dimensional muscle synergy analysis method sensitively captured improvements in neuromuscular coordination during rehabilitation, aligning well with traditional clinical evaluation results. Additionally, the proposed CMFI fatigue-monitoring method proved capable of real-time fatigue assessment and training intensity adjustment, significantly enhancing training safety and rehabilitation effectiveness. These results demonstrate that the proposed methods provide objective, quantitative, and practical tools for rehabilitation evaluation and control, offering important technical foundations for the further development and clinical application of piano-based rehabilitation devices. Future work will focus on expanding the sample size, optimizing model personalization strategies, and integrating closed-loop adaptive training systems based on real-time biomechanical and neuromuscular feedback.

## Figures and Tables

**Table 1:** Baseline characteristics table.

Finger	MP/%	PP/%	DP/%
Thumb	8.36	-	6.22
Index finger	4.55	5.19	6.11
Middle finger	7.39	9.03	9.87
Ring finger	7.24	8.46	14.25
Little finger	8.76	11.72	25.98

## Data Availability

Details of data availability are available from the first author on request.
